# Case Report: Complete Remission of C1q Nephropathy Treated With a Single Low-Dose Rituximab, a Reality or Coincidence?

**DOI:** 10.3389/fped.2020.568773

**Published:** 2021-02-04

**Authors:** Rui Ma, Dengyan Wu, Zhiqin He, Qian Chang, Yonghong Yang

**Affiliations:** ^1^Department of Pediatric Nephrology, Lanzhou University Second Hospital, Lanzhou, China; ^2^Department of Nephrology, Gansu Children's Hospital, Lanzhou, China; ^3^Department of Pediatrics, Children's Hospital of Xi'an International Medical Center, Xi'an, China

**Keywords:** C1q nephropathy, nephrotic syndrome, rituximab, complete remission, B lymphocyte depletion

## Abstract

C1q nephropathy is a glomerulopathy that is characterized by large amount of C1q deposits in the glomerular mesangium. It is a diagnosis of exclusion after ruling out systemic lupus erythematosus and membranoproliferative glomerulonephritis by systemic and serological examination. The pathogenesis of C1q nephropathy is unclear. In addition, there is very little generalizability in the treatment and prognosis for pediatric C1q nephropathy due to diversities in clinical manifestations and pathological types. Rituximab is a human/mouse chimeric monoclonal antibody against CD20, which is primarily used for treating lymphomas and, most recently, has been used to treat certain kidney diseases including C1q nephropathy. In this report, we used one quarter of the typical dose of rituximab for lymphoma treatment to achieve complete remission in a C1q nephropathy patient, significantly reducing deposition of immune complexes and glomerular damage. This case indicates that dosage reconsiderations may be necessary for rituximab in treatment of pediatric C1q nephropathy.

## Introduction

C1q nephropathy is characterized by large amounts of mesangial immunoglobulin and complement deposition with predominant appearance of C1q, after exclusion of systemic lupus erythematosus and membranoproliferative disease (1). The incidence of C1q nephropathy varies in different reports ranging from 0.2 to 16% with no gender differences (1–4). It is speculated that complement activation and glomerular antigen–antibody complex formation underlie pathogenesis of C1q nephropathy, with additional involvement of alternative complement pathway and lectin pathway (5).

The clinical manifestations of C1q nephropathy are diverse. Most of the patients present with nephrotic syndrome, some with acute or chronic glomerulonephritis, and, occasionally, the only presentation may be hematuria. The disease also presents with various nephropathologies, the most common being minimal change nephropathy, focal segmental glomerulosclerosis nephropathy, and immune complex-mediated mesangial proliferative glomerulonephritis (5). These distinctions are especially important, as prognosis of C1q nephropathy in patients depends on their pathological type and clinical manifestations. Notably, children with C1q nephropathy have higher rate of recurrence and shorter recurrence interval compared to nephrotic syndrome without C1q deposition (6). There are no disease-specific treatment guidelines for C1q nephropathy. Currently, treatment guideline for C1q nephropathy follows the general guidelines for treatment of primary nephrotic syndrome, with corticosteroid as first-line treatment. If a patient is corticosteroid-dependent or corticosteroid-resistant, a second-line medication, e.g., cyclosporine, is selected with criteria based on patient condition. Of interest to the case presented here, a small number of cases have reported the efficacy of rituximab for treating C1q nephropathy, with significant improvement of renal function and clinical manifestations (7–10). Rituximab is a human/mouse chimeric monoclonal antibody targeting CD20 (11). It was originally used to treat B-cell non-Hodgkin's lymphoma but has since been widely used in autoimmune anemia, rheumatic diseases, and more recently used for the treatment of autoantibody-related kidney diseases, including antineutrophil cytoplasmic antibodies (ANCA)-associated nephritis and membranous nephropathy (12–14). The target of rituximab is CD20, a tetra-transmembrane protein expressed in pre-B cells. Rituximab depletes B cells via direct signal-induced apoptosis, complement-dependent cytotoxicity, antibody-dependent cell-mediated cytotoxicity, and antibody-dependent phagocytosis (11, 12, 14). Depletion of B cells results in the reduction in antibody and immune complex formation and, ultimately, reduces C1q deposition.

The standard dosage of rituximab for treating nephrotic syndrome is adapted from existing guidelines for lymphoma treatment (10, 12, 15, 16). No disease-specific guidelines has been made available for treatment of C1q nephropathy using rituximab. In this report, we show complete remission achieved via a significantly reduced dose of rituximab in a pediatric patient with C1q nephropathy. Our results suggest the need for further investigation into disease-specific dosage for C1q nephropathy, especially in the pediatric setting, with benefits of lower risks of adverse events as well as cost considerations in uninsured and underinsured patients.

## Case Presentation

**Figure 1:**
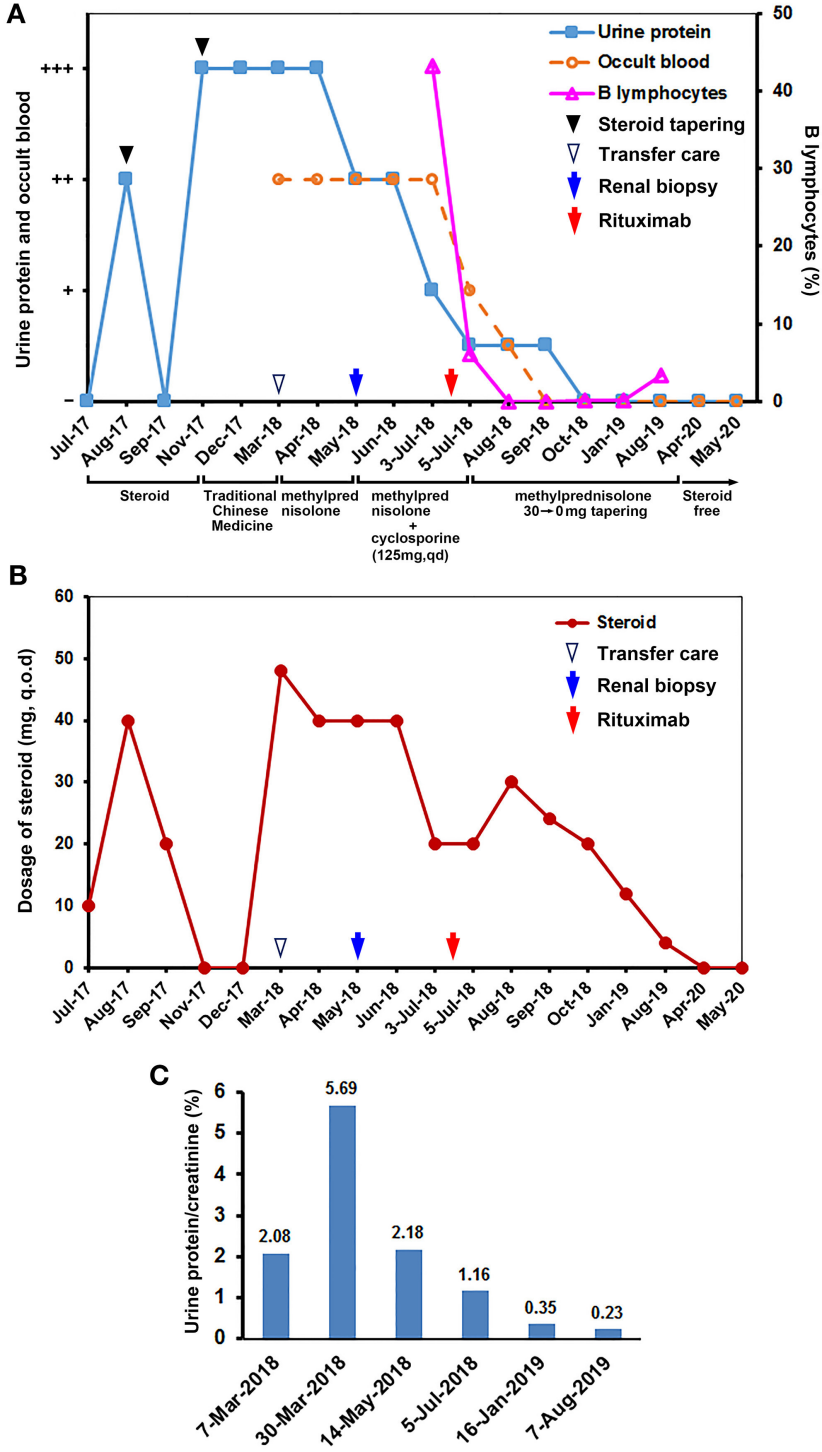
The clinical course for the C1q nephropathy patient. **(A)** Selected urinalysis results and clinical course for the patient. Proteinuria and urinary occult blood have been negative for >18 months since administration of rituximab, indicating long-term remission. No relapse occurs after withdrawing maintenance dose of corticosteroid. **(B)** The course of steroid treatment is shown for corresponding time points in **(A)**. **(C)** Urinary protein/creatinine ratios during the care provided by us.

The patient is a 7-year-old Asian girl who was diagnosed with nephrotic syndrome in June 2017 in a local primary care clinic before transferring care to our department 9 months later. Prior to diagnosis, patient was healthy, with no significant birth history and no history of surgery, trauma, or blood transfusion. The patient visited the local clinic due to cold/flu-like symptoms, swelling eyelids and lower limbs, and abdominal pain that was eventually diagnosed as nephrotic syndrome. No family history was reported. Based on the records provided by the patient's parents, the patient underwent a course of oral corticosteroid, and subsequently, urine protein was negative, indicating corticosteroid-sensitive response. However, patient's urine protein increased once again after tapering corticosteroid, and her steroid dosage was increased. Subsequently, her urinalysis was once again negative for protein, but each time steroid taper was attempted, patient relapsed with significant proteinuria. Although her disease was progressively steroid dependent, corticosteroid was discontinued per the request of the patient's parents after several courses due to concern for adverse effects of long-term therapy. Instead, the patient was switched to traditional Chinese medicine (ingredients unknown) for nearly 4 months (Figure 1A). During the treatment with traditional Chinese medicine alone, the patient visited her local primary care clinic for several urinalyses that consistently demonstrated 3+ proteinuria and occult blood. Meanwhile, the patient showed progressive clinical decline with severe complications including urinary tract infections, systemic edema, shortness of breath, frequent urination, and dysuria. Due to significant disease progression, patient was referred to our hospital in March 2018.

On presentation to our hospital, the patient was admitted to the Pediatric Intensive Care Unit (PICU) and found to have a fungal infection, heart failure, hypertension, ascites, and persistent oliguria. Routine examination and laboratory tests showed body weight of 33 kg, body surface area of 1.11 m^2^, serum albumin of 14.3 g/L, total cholesterol of 20.78 mol/L, and triglyceride of 11.13 mmol/L, indicating hypoproteinemia and hyperlipidemia. Renal function tests showed serum uric acid of 545 μmol/L, creatinine of 121.0 μmol/L, blood urea nitrogen (BUN) of 22.4 mmol/L, and glomerular filtration rate (GFR) of 37.86 ml/min, indicating azotemia and renal dysfunction. Initial treatment included correcting electrolyte disorder, diuresis, anticoaglutant therapy, and fluconazole (3.6 mg/kg) for fungal treatment. During this time, she was also treated with methylprednisolone (2 mg/kg daily) and aldehyde oxystarch, without significant efficacy (Figures 1A,B). Therefore, the patient underwent continuous renal replacement therapy (CRRT) with significant improvement of disease. Laboratory tests showed blood creatinine of 46 μmol/L, BUN of 10 mmol/L, and GFR of 99.59 ml/min. Patient was then transferred from PICU to our department for subsequent therapy. Autoimmune antibody panel was ordered, and all resulted negative. These included antinuclear antibody (ANA), antidouble-stranded DNA (anti-dsDNA), anti-Smith, antihistone antibody (AHA), anti-Sjögren's-syndrome-related antigen A (anti-SSA), anti-SSB, rheumatoid factor, ANCA, cardiolipin autoantibody, antimitochondrial antibody (AMA), smooth muscle antibody (SMA), anticentromere antibody, and antibasement membrane antibody. With negative immunological workup, we recommended a renal biopsy, but this was declined by her parents at that time with concerns regarding invasiveness of biopsy. During that admission, patient experienced repeated hypertension that was difficult to control on nifedipine and, subsequently, developed convulsions accompanied by right hemiplegia concerning for hypertensive encephalopathy. She subsequently underwent aggressive sodium nitroprusside (1–5 μg/kg/min), diuretic (spironolactone 20 mg and hydrochlorothiazide 10 mg daily), and steroid therapy (methylprednisolone 40 mg, q.o.d). Following these, her blood pressure returned to normal, neurological symptoms resolved, and systemic edema also improved. Laboratory tests showed that urine protein and occult blood decreased to 2+, blood creatinine was 42.0 μmol/L, and hypoproteinemia was corrected. Patient was discharged with continuation on methylprednisolone (40 mg daily in divided doses), losartan potassium (20 mg daily), and spironolactone (20 mg daily).

**Figure 2:**
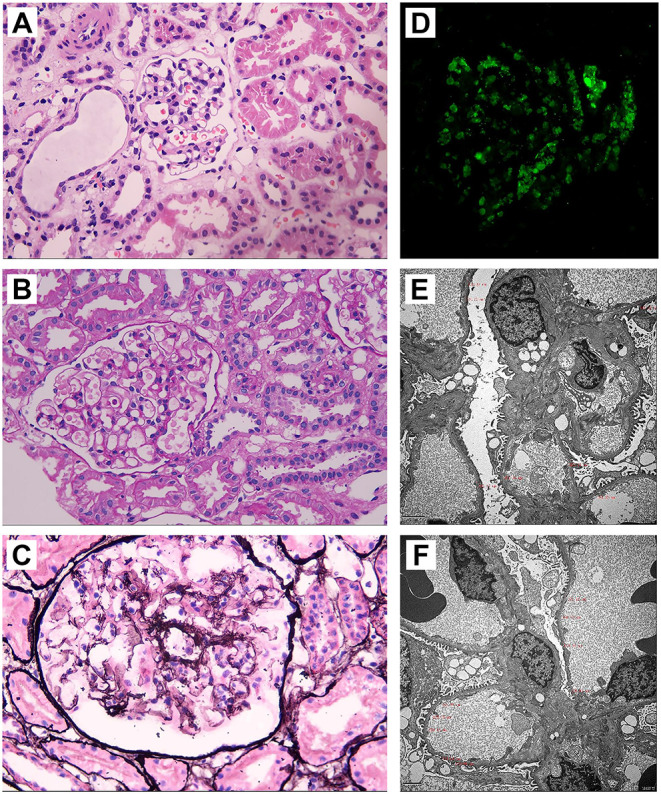
Pathology evaluation for the C1q nephropathy patient. **(A)** Hematoxylin–eosin staining shows increased number of cells in glomerulus. No glomerular crescent, focal segmental glomerular sclerosis, and fibrosis are noted. However, renal tubule dilation and vacuolar degeneration of epithelial cells are present, and renal interstitium is slightly infiltrated with inflammatory cells, indicating a minimal change disease (MCD). **(B)** Periodic acid–Schiff (PAS) staining shows no remarkable cellular proliferation of mesangium and stroma. **(C)** Periodic Schiff–methenamine (PASM) staining shows normal capillary loops and no significantly thickened basement membrane. **(D)** Immunofluorescent staining confirms large amount of C1q deposition. **(E,F)** Transmission electron microscopy shows vacuolar degeneration of epithelial cells, podocyte foot processes effacement, proliferation of mesangium and stroma, and thin basement membrane.

During her outpatient follow-up, patient's urine protein consistently returned 2–3+, suggesting steroid resistance. The need for tissue diagnosis via biopsy was reiterated, but patient's family continued to refuse at that time. Therefore, cyclosporine 5 mg/kg PO was added to her regimen. Subsequent labs showed improved proteinuria without significant decrease in serum creatinine despite the addition of cyclosporine. Due to frequency of relapses, renal biopsy was discussed again with the patient's parents for which consent was given in May 2018, and microscopy results were consistent with C1q nephropathy (Figures 2A–F).

Based on the pathological findings, a trial of rituximab was initiated after lymphocyte subset analysis showed B lymphocyte ratio of 43.2%. The patient received a single intravenous dose of 100 mg rituximab (93 mg/m^2^). During infusion, patient experienced facial flushing but no other significant discomfort. B lymphocyte subset fell to 6.1% after 1 day. After 2 weeks of rituximab treatment, B cells were completely depleted, and renal function was significantly improved with a GFR of 93 ml/min (Figure 1A). Patient was then given prednisone starting at 30 mg every other day and gradually tapered at a rate of 5 mg/month. At 1-year follow-up in August 2019, patient presented with symptoms of urinary tract infection. Urinalysis at that time showed 4+ white blood cells (WBCs) but was negative for protein and blood. Serum tests showed normal kidney function and 3.30% of total B lymphocyte subset. At present, patient has maintained normal kidney function off steroid therapy and has had no relapses since rituximab therapy. The treatment courses and selected laboratory results are shown in Figure 1.

## Discussion

Although C1q nephropathy currently has no discrete clinical considerations from nephrotic syndrome, its pathogenesis and disease course do indicate disparate treatment guidelines. Patients with C1q nephropathy are frequently steroid dependent, more likely to relapse, and have a shorter recurrence period than nephrotic syndrome patients with no C1q deposition (4).

Typically, pathological findings in children with nephrotic syndrome are mostly small lesions, and in these cases, steroid therapy is preferred. If treatment is inefficacious, an immunosuppressor is added, with risk of detriment to renal function. As aforementioned, rituximab has been used to treat C1q nephropathy and shown to partially restore kidney function (9). At present, the dose of rituximab for treating C1q nephropathy is based on guidelines for lymphoma treatment, i.e., four doses of 375 mg/m^2^ at weekly intervals (7). Recent studies reported complete remission for patients with C1q nephropathy by using a single dose or two doses of rituximab at 375 mg/m^2^ (8, 10). In malignant disease such as lymphoma, abnormal lymphocytes are generally significantly elevated, while the number of lymphocytes in C1q nephropathy does not have similar magnitude of elevation. Therefore, we posited that the dosage of rituximab for treating C1q nephropathy may not need to be as high as that for lymphoma. Several studies have used low-dose rituximab (a fixed dose of 100 mg) to treat adult patients with idiopathic autoimmune hemolytic anemia and primary immune thrombocytopenia and showed comparable efficacy of standard dose (17, 18).

In this case, we reported the first successful use of a single low dose of rituximab (100 mg or 93 mg/m^2^) for C1q nephropathy treatment, a quarter that of the dosage indicated for lymphoma. After treatment, our patient has shown normal renal function, negative urine protein, and occult blood for >18 months and is currently steroid-free, suggesting that successful remission was achieved at this significantly reduced dosage. The side effects of rituximab have not been clarified, with some occurring early in administration, including nausea, rash, and bronchospasm, although adverse effects can be minimized by controlling infusion rate (15). Viral infections and pyelonephritis accompanied by neutropenia can also occur during the rituximab treatment (15). As shown in the literature, low-dose rituximab may reduce risks of severe side effects such as neutropenia and pulmonary toxicity (19). A lower dose can also be cost saving and shortens infusion time, which are important considerations in making biologics accessible to indigent populations. In the setting of good treatment efficacy with better controlled adverse effect profile, reduced dosage treatment with rituximab in C1q nephropathy warrants additional examination.

Our report features only one case, which is a limitation presented by the rarity of C1q nephropathy in our practice. Although the response of this patient to such a low dose of rituximab may occur on occasion and could be race dependent, the overall outcomes for this patient is remarkable. These results suggest that the dosage of rituximab for treating pediatric nephrotic syndrome, particularly C1q nephropathy, may be significantly reduced. To further investigate, a prospective case-controlled clinical trial recruiting larger, more diverse populations will be needed.

Although the pathogenesis of C1q nephropathy still remains unclear, it is speculated that complement activation and glomerular antigen–antibody complex formation play a central role. Studies show that C1q molecules have affinity to various substances including DNA, RNA, viral proteins, Gram-negative bacteria, and various immune cells (5). C1q has been demonstrated to enhance B-cell response to antigens, which underlies the reason for use of rituximab to deplete B cells and reduce deposition of immune complexes. Previous studies have suggested that dosage of rituximab should be determined according to serum CD19 level, which is expressed in pre-B cells prior to differentiation (10). This method of quantitatively metered administration may be a focus of clinical trials in the future. Some Literatures also report that rituximab is less effective in patients with hormones and cyclosporine-dependent nephrotic syndrome (12). In consideration of increased complications with multidrug treatment, administration of rituximab in the early stages for steroid-dependent patients can effectively prevent progression of disease while also mitigating the myriad of harmful side effects of steroid treatment. There are also some reports suggesting that subcutaneous injection of rituximab is more time saving and less labor intensive compared to intravenous administration (11, 13). In addition, rituximab can be subcutaneously injected with recombinant human hyaluronidase that increases the dispersion and absorption of rituximab (11). Given the multitude of areas for development, we expect to continue investigating the mode of administration and dosage of rituximab to achieve maximum efficacy and minimize adverse outcomes.

## Data Availability Statement

The original contributions presented in the study are included in the article/supplementary materials, further inquiries can be directed to the corresponding author/s.

## Ethics Statement

The studies involving human participants were reviewed and approved by the Ethical Committee of Lanzhou University Second Hospital. Written informed consent to participate in this study was provided by the participants' legal guardian/next of kin.

## Author Contributions

RM collected the data and prepared the manuscript. DW participated in the patient's care. ZH collected and analyzed the data. QC participated in the patient's clinical care. YY participated in the patient's care, supervised the study, analyzed the data, and wrote the manuscript. All authors contributed to the article and approved the submitted version.

## Conflict of Interest

The authors declare that the research was conducted in the absence of any commercial or financial relationships that could be construed as a potential conflict of interest.
